# School-based girls' clubs as a means of addressing sexual and gender-based violence in Swaziland

**DOI:** 10.1186/1753-6561-9-S4-A5

**Published:** 2015-07-07

**Authors:** Cebile Manzini-Henwood, Nokwanda Dlamini, Francis Obare

**Affiliations:** 1 Swaziland Action Group Against Abuse, P.O. Box 560, Matsapha, Swaziland; 2 Population Council, P.O. Box 17643-00500, Nairobi, Kenya

## Background

Sexual and gender based violence (SGBV) is a global health and human rights problem which is of particular concern in sub-Saharan Africa because of the compounding effects of high HIV prevalence. In Swaziland - a small, land-locked country of just over one million people - women and girls face the disproportionate burden of both SGBV and HIV: Nearly half (48%) of those aged 13-24 years reported having experienced some form of sexual violence (including rape, threat of rape, unwanted touching or groping), and among those of secondary school age (13-17), only 37% reported that their first sexual experience was voluntary [1]. Although available evidence suggests that most SGBV cases occur in the home or community, schools are not entirely safe places for girls. For example, findings from a national survey on violence against children in Swaziland indicate that among incidents of sexual violence experienced before age 18 in Swaziland, 10% occurred at school and another 10% on the way to and from school [1].

There is growing evidence of direct and indirect links between SGBV and HIV, with violence being both a cause and outcome of HIV infection [2,3]. The national HIV prevalence among adults (15-49) in Swaziland is among the highest in the world, at 26% [4]. This study aimed to assess the effectiveness of a girls' empowerment intervention in regard to changing in-school girls' knowledge, attitudes, and practices related to SGBV.

## Materials and methods

### Study design

The study used a pre- and post-intervention design without a comparison group and included three co-educational secondary day schools in the Lubombo Region of Eastern Swaziland.

### Intervention

The intervention was implemented over a 12-month period from July 2012 to July 2013, and involved three main activities, namely: revising existing girls' club resources, training club mentors, and running weekly, SGBV-focused club sessions with girls in school. Existing girls' empowerment club resources of the Swaziland Action Group Against Abuse (SWAGAA) were revised to ensure an emphasis on SGBV. Specifically, SWAGAA's training curriculum (used to train girls' club mentors) and training manuals (used by trained mentors to facilitate girls' club sessions) were amended to incorporate an 'asset-building approach' [5] for girls (particularly focused on building girls' social assets, such as friendship and participation in extra-curricular activities), and to include comprehensive information on gender and SGBV; how to identify a risky situation; and how to report incidents of violence.

The revised training curriculum was used to train mentors on their roles and responsibilities over a three-day period. A total of 15 female mentors aged 18-25 years were recruited from the communities surrounding the participating schools. Their training focused on an overview of SGBV and its relevance for girls, modalities for recruiting girls for the clubs and for handling SGBV reporting and referrals, and on SWAGAA programs and resources. Mentors were responsible for running the girls' clubs once a week. They reported directly to SWAGAA and were compensated for their time based on local rates for equivalent work.

In each school site, all girls aged 16 and above were invited to participate in the weekly girls' clubs, which were structured around groups of about 20 girls per club. Club sessions took place in a pre-identified 'safe space' (in this case, a class room). Club activities included interactive discussions facilitated by a mentor and guided by the revised club manual.

### Data collection and analysis

Data collection involved baseline and endline self-administered quantitative interviews with in-school girls ages 16 and above who participated in the girls' clubs. These data were entered in Excel and analyzed using STATA and Excel. Analysis entailed simple frequencies, percentages and cross-tabulations with Chi-square tests, as well as significance tests of proportions, and a comparison of baseline and endline results.

## Results

At both baseline (n = 247) and endline (n = 143), the majority of students were aged 16-18 years, were in Form 4 (the 4^th ^year of a five-year secondary school), had two living parents, and had no sexual partner.

The effectiveness of the SGBV-focused girls' clubs is examined in terms of changes between baseline and endline in girls': 1) social assets; 2) awareness about SGBV; 3) practices and experiences related to SGBV; and 4) attitudes towards SGBV. Results around these four areas are summarized below.

### Social Assets

There were significant increases between baseline and endline in the proportions of girls that reported that they were engaged in an income-generating activity (from 6% to 13%), had many friends in the neighborhood (from 41% to 52%), had two or more close friends they could confide in (from 60% to 74%), or that they were taking part in extra curricula activities (from 68% to 100%).

### Awareness about SGBV

There were significant improvements in the levels of SGBV awareness among girls. The proportion of students who reported the following events or experiences significantly increased between baseline and endline: other girls in their school being teased or subjected to verbal sexual harassment (from 24% to 38%); personally being subjected to sexual comments by fellow students at their school (from 19% to 34%); and ever experiencing any form of SGBV either at school or in the community (from 50% to 67%).

### SGBV-Related Practices

The proportion of girls that indicated they would report incidents of sexual comments by another student to authority figures (teachers, the school principal, or the police) significantly increased from 41% at baseline to 57% at endline. However, there was no significant change in the proportion of girls that indicated that they would report sexual comments by a teacher to authority figures (from 38% at baseline to 40% at endline). Nor was there any significant change in the proportion of girls that reported they would decline sexual advances from a student (69% at baseline and a similar proportion at endline) or from a teacher (47% at baseline and 43% at endline).

### Attitudes toward SGBV

There were significant positive changes in the attitudes of girls regarding SGBV. Girls' responses in this area indicated significant positive changes in 13 out of a total of 21 items used to measure attitudes towards SGBV. The changes were characterized by significant increases between baseline and endline in the proportions of students that disagreed with statements that justified SGBV, as well as significant increases in the proportions that agreed with statements promoting the rights of women and girls in society.

## Conclusions

The study findings show high levels of reported abuse among in-school girls in Swaziland, and demonstrate the overall ability of the intervention model to contribute to improved SGBV outcomes among this population. While the SGBV-focused girls' clubs were effective in improving girls' social assets, increasing their awareness, and positively changing their attitudes towards, and potential practices against, SGBV, they were less successful in improving the odds that girls would decline sexual advances from fellow students or teachers. This suggests that the interventions could further be strengthened by incorporating components aimed at enhancing self-efficacy among girls. With new funding, the Swaziland Action Group Against Abuse is currently working on enhancing this intervention by incorporating a self-efficacy component into the girls' clubs.
